# Drug clinical trials on high-grade gliomas: challenges and hopes

**DOI:** 10.20892/j.issn.2095-3941.2023.0364

**Published:** 2024-02-05

**Authors:** Mengqian Huang, Shenglan Li, Parker Li, Zhuang Kang, Botao Zhang, Wenbin Li

**Affiliations:** 1 Cancer Center, Beijing Tiantan Hospital, Capital Medical University, Beijing, China; 2 Clinical Medicine, Shanghai Jiao Tong University School of Medicine, Shanghai, China

Gliomas are the most common group of malignant central nervous system tumors across all ages and have high heterogeneity. According to histopathologic criteria and molecular pathology, gliomas are classified as grades 1–4. High-grade gliomas (HGG), including the most aggressive subtype, glioblastoma (GBM), belong to grade 3 or 4. Although a standard therapy comprising surgical resection, chemotherapy, and radiation is available, almost all patients with HGG experience recurrence within several months and a dismal prognosis^1^. Owing to the aggressive growth pattern of HGG, complete surgical resection is difficult to achieve, particularly for deep-seated tumors, and recurrence is inevitable. Therefore, systematic treatment approaches are urgently needed.

In recent years, clinical research on glioma medications has made remarkable breakthroughs in many areas. One notable area of focus is the advancement of immunotherapies. In investigation of the molecular characteristics of glioma cells, targeted therapies have become a research focus. Various drugs targeting particular molecules or signaling pathways have been developed. These medications can accurately target the growth and spread of tumor cells while causing less harm to normal brain tissues than to the tumor cells.

## Challenges

Systematic treatment approaches, which are represented by drugs, face several challenges. HGGs exhibit high inter- and intra-tumor heterogeneity with complex molecular characteristics, thus leading to difficulties in the development of safe and effective therapies. Another major obstacle is the emergence of drug resistance, which may be associated with the subpopulations of glioma stem cells. In addition, the blood–brain barrier (BBB) impedes drug absorption. Failure to deliver drugs across the BBB leads to insufficient therapeutic drug concentrations at the brain tumor site, thus hampering effective treatment^2^. Therefore, additional efforts should be made to penetrate the BBB to enhance drug efficacy.

To date, no therapy has demonstrated curative potential. Therefore, a need exists to explore novel therapeutics.

## Hopes

### Chemotherapy

Efforts have been dedicated to the development of traditional chemotherapy. The front-line drug temozolomide (TMZ) from the Stupp regimen has long played a major role in chemotherapy. Challenges with chemotherapy drugs involve increasing their BBB permeation while minimizing their toxicity to normal tissues.

Some nitrosoureas have antitumor activity and high lipophilicity, thus allowing them to permeate the BBB. Hence, they have potential for treating gliomas. Lomustine (CCNU) is a member of the first-generation nitrosoureas and a key part of the PCV regimen (where P indicates procarbazine, C indicates lomustine, and V indicates vincristine) used in recurrent gliomas. CCNU has served as the control arm in several randomized phase III trials. Patients with MGMT-methylated tumors may benefit from nitrosoureas, although no survival advantages have been observed with nitrosoureas combined with bevacizumab^3^.

Irinotecan is a topoisomerase 1 inhibitor that has shown anti-glioma efficacy in animal studies. Owing to its lipophilic characteristics, irinotecan is an attractive drug that can be combined with other therapies, such as TMZ or antivascular endothelial growth factor (VEGF) therapies. A phase II clinical study conducted in 2007 used a combination of irinotecan and bevacizumab for the treatment of gliomas and yielded favorable results^4^.

### Immunotherapy

Several studies have highlighted the immunosuppressive characteristics of gliomas with elevated levels of immunosuppressive factors and decreased immune cell activity. Treating gliomas through immune system activation has emerged as a promising new approach for improving patient survival and quality of life.

The exploration of immune checkpoints, such as programmed cell death protein 1(PD-1)/programmed death-ligand 1(PD-L1), plays a crucial role in the advancement of cancer immunotherapy. However, clinical trials on PD-1/PD-L1 in recurrent or newly diagnosed GBM have not indicated its advantages in either overall survival (OS) or progression-free survival (PFS)^5^. The lack of meaningful benefit is attributed to the influence of the innate immunosuppressive microenvironment. A rational combination of trials integrating preclinical and clinical investigations may be suitable for investigating this drug. A study has found that anti-PD-1 combined with anti-PD-L1 exhibits intracranial efficacy in patients with melanoma with brain metastases; although this activity is attributable partly to BBB dysfunction caused by tumor metastasis, this new approach may aid in development of the next generation of glioma clinical trials^6^. A phase I clinical trial has investigated this combination treatment, and demonstrated its safety and benefits in OS in recurrent GBM (including intravenous and intracranial administration)^7^, and phase II/III studies are currently in progress (ClinicalTrials.gov identifiers NCT04145115 and NCT04396860).

Oncolytic viruses (OVs) are products of genetic engineering designed to selectively infect and kill tumor cells, while causing minimal harm to normal tissues. Beyond their direct oncolytic effects, OVs upregulate immunosuppressive metabolites in the tumor microenvironment and modulate the expression of checkpoint proteins, thus leading to a sustained immune response. Hence, OVs are highly promising therapeutic approaches that are being extensively studied preclinically and clinically^8^.

Herpes simplex virus (HSV) is neurotropic and is ideal for neurogenic tumor targeting. HSV-1 was the first genetically engineered virus to be used in treating malignant glioma; however, during the research period, neither its safety nor its efficacy was confirmed. Since then, mutant HSV vectors have been developed, including the first generation represented by G207, with enhanced safety^9^; the second generation represented by T-vec, the first OV whose efficacy was confirmed in a phase III clinical trial^10^; and the third generation oncolytic HSV-1, represented by G47∆.

With a triple mutation in the genome, G47Δ displays greater replicative ability and higher anti-tumor efficacy than G207, and is an effective human GBM stem cell killer^11^. After the confirmation of its safety in a phase I/II study in patients with recurrent GBM, a phase II clinical trial further demonstrated its effectiveness in 2 cases (15.4%) maintaining stable disease^12^. Therefore, G47Δ was designated as a SAKIGAKE (breakthrough therapy) product and approved for malignant glioma treatment in Japan.

Another focus of research is delta-24-arginine-glycine-aspartate (delta-24-RGD or DNX-2401), a second-generation oncolytic adenovirus that has been demonstrated to have anti-glioma activity in preclinical studies^13^. The completed phase I clinical trial on DNX-2401 has yielded positive outcomes: some patients experienced tumor regression after intratumoral injection of DNX-2401. Biological evaluation of the posttreatment tumor tissues has suggested that the antitumor effect may stem from the direct oncolytic effects of viral infection and the activation of immune-mediated anti-glioma responses^14^. Although OVs alone have demonstrated potential, their clinical benefits remain limited. Combined treatment with OVs may enable high efficacy. A phase II clinical trial has recently been completed and has yielded encouraging outcomes^15^. The study evaluated the efficacy of a combination treatment of immune checkpoint inhibitors and OVs in gliomas—a therapy that had been demonstrated to be effective in other tumors—and observed durable responses^15,16^.

On the basis of the results of ongoing and completed clinical trials, OVs have potential for clinical use with favorable safety profiles in glioma treatment. Nevertheless, several challenges must be overcome. Although intratumor injection can achieve maximum delivery to tumors with minimum systematic toxicity, a neurosurgical procedure is inevitable and carries several risks, such as intracranial infection.

OH2, a promising OV derived from HSV-2, was designed to decrease toxicity and increase tumor selectivity by deleting the ICP34.5 neurovirulence gene; it has enhanced antitumor immunity through the addition of the human granulocyte macrophage colony-stimulating factor (GM-CSF) gene^17^. The tolerance to, and antitumor activity of, OH2 have been confirmed in a phase I/II clinical trial in patients with advanced solid tumors^18^. A clinical trial in gliomas is ongoing (ClinicalTrials.gov identifier NCT05235074). In contrast to other OV clinical trials, the OH2 trial used a protocol including an Ommaya reservoir placement surgery, with the goal of decreasing potential risks during subsequent drug administration. This innovative approach has provided remarkable inspiration for OV clinical trials that require intratumoral injection in patients.

Cellular vaccines are a viable option for glioma immunotherapy. The dendritic cell vaccine (DCV) is composed of dendritic cells loaded with tumor antigens capable of provoking immune responses. The relative safety and potential efficacy of DC-based vaccinations have been observed in phase I/II clinical trials^19^. A completed large phase III non-randomized controlled trial in 331 GBM patients has demonstrated the feasibility and potency of tumor lysate-loaded DCV (DCVax-L)^20^. The study has established the safety profile and demonstrated a survival benefit of the combination therapy, on the basis of an OS surpassing that of patients receiving the standard of care (SOC). Patients receiving the combined therapy exhibited a prolonged median OS (22.3 months after surgery in patients with newly diagnosed GBM and 13.2 months after relapse in patients with recurrent GBM); therefore, DCVax-L has substantial potential in the treatment in GBM.

Some studies have focused on small molecular compounds with antitumor potential. One of these compounds is chlorogenic acid (5-caffeoylquinic acid, CGA), a phenolic compound isolated from fruits and plants, such as Eucommia ulmoides or Flos Lonicera. In a study on depression, the observation of CGA’s ability to cross the BBB has led to its application in brain tumor treatment^21^. Preclinical research has also investigated its anti-tumor mechanism, focusing primarily on its effect on immunoregulation. A study on macrophage polarization has indicated that CGA leads to the conversion of M2 macrophages, which predominate in the tumor microenvironment, into M1 macrophages, which induce tumor dissolution, thereby limiting glioma growth^22^. These promising results supported a phase I clinical trial evaluating the application of CGA in recurrent GBM. This completed trial has established the safety profile of CGA and its potential OS benefit^23^. A phase II trial is currently underway to further investigate its efficacy (ClinicalTrials.gov identifier NCT03758014).

### Targeted therapy

Extensive research has been conducted on targeted therapy, which offers major advantages, such as specificity and selectivity. A compound of interest is procaspase-activating compound-1 (PAC-1), which specifically targets procaspase-3 (PC-3) by chelating inhibitory labile zinc ions. PC-3 is overexpressed in GBM and shows potential oncogenic effects; moreover, its cleavage into caspase-3 plays a critical role in apoptosis. Therefore, researchers are investigating PC-3 activation as a novel anticancer target. A phase I study of PAC-1 for advanced malignancy treatment has established the maximum tolerated dose of PAC-1 and identified its clinical activity, particularly in neuroendocrine tumors^24^. On the basis of that study and hypothesis-generating preclinical data regarding gliomas, including the ability of PAC-1 to cross the BBB in a mouse model, a phase I study of PAC-1 in combination with TMZ was conducted on patients with HGG. The results have demonstrated that the combination is safe and well tolerated, and thus warrants further investigation^25^.

Small molecular inhibitors targeting cyclin-dependent kinases 4 and 6 (CDK4/6) are also under investigation. CDK4/6 deregulation has been observed in gliomas, and inhibition of CDK4/6 arrests the cell cycle in the G1 phase, thus inhibiting tumor cell proliferation. CDK4/6 inhibitors have shown efficacy in certain tumors, and 3 inhibitors (ribociclib, palbociclib, and abemaciclib) have been approved by the U.S. Food & Drug Administration (FDA). A phase I/II study has found that ribociclib is safe and has promising primary efficacy when used after radiotherapy in children with newly diagnosed diffuse intrinsic pontine glioma^26^. Some trials have been performed on novel CDK4/6 inhibitors in the early phase (registration number ACTRN12621000479808). Resistance to CDK4/6 inhibitors remains a hurdle to be overcome, and a recent study has suggested that combining these inhibitors with tyrosine kinase inhibitors (TKIs) may yield consistent tumor responses and serve as a potential new strategy^27^.

TKIs are a prevalent research topic, particularly in relation to epidermal growth factor receptor (EGFR). EGFR mutation is a potential marker for glioma treatment and is associated with tumor proliferation and migration. Osimertinib, which has better BBB penetration ability than first- and second-generation EGFR inhibitors, shows potential in anti-GBM therapy^28^. Bevacizumab targets VEGF, thereby inhibiting angiogenesis. Although a clear survival benefit is lacking, this treatment prolongs PFS, decreases peritumoral edema, and enables lower steroid use. Regorafenib is a multitargeted TKI drug that has shown an encouraging OS benefit in recurrent GBM; the AGILE trial (ClinicalTrials.gov identifier NCT03970447) will further investigate the role of this treatment in patients with newly diagnosed GBM^29^.

Another reliable marker for targeted therapy is the V-RAF murine sarcoma viral oncogene homolog B1 (BRAF) mutation. The efficacy of BRAF-targeted therapy, such as dabrafenib, has been established in other cancers^30^. Despite the relatively low incidence of BRAF mutation in HGG, clinical trials have supported further investigation in patients with HGG with BRAF mutation^31^.

Isocitrate dehydrogenase (IDH) is a common molecular marker in tumors. Inhibitors such as ivosidenib and vorasidenib have exhibited promising efficacy in treating low-grade glioma with mutant IDH^32,33^. Notably, whereas drugs that target mutant forms of IDH are focused primarily on low-grade glioma, the key clinical trials started before the WHO 2021 classification. Therefore, patients whose diagnoses previously met the eligibility criteria may now carry an updated classification of HGG by histology and molecular analyses. On the basis of the results of these studies, clinical trials for IDH-mutated HGGs are ongoing (NCT05484622 and CTR20211073).

Mutations in the PI3K/AKT/mTOR pathway are common among patients with IDH wild-type GBM^34^. Nevertheless, the efficacy and safety of mTOR inhibitors, used either alone or in combination with other treatments, have not met expectations, possibly as a result of the complex molecular regulation of this pathway^35,36^.

**Table 1:** Selected drug clinical trials in patients with HGG

Therapy	Intervention	Phase	Tumor type	Summary of results/status	Year	Reference	Trial number
Chemotherapy	CCNU *vs.* CCNU + bevacizumab	III	Progressive GBM	Median PFS 1.5 months *vs.* 3.8 months	2017	^3^	NCT01290939
	Irinotecan + bevacizumab	II	Recurrent HGG	For patients with GBM: median PFS 20 weeks; median OS 40 weeks	2007	^4^	NCT00268359
Immunotherapy	2 pembrolizumab doses before surgery and every 3 weeks afterward	II	Recurrent (operable) GBM	Median PFS 4.5 months; median OS 20 months	2020	^5^	NCT02337686
	Neoadjuvant nivolumab and ipilimumab	I	Recurrent GBM	Median OS 38 weeks	2021	^7^	NCT03233152
	Ipilimumab and nivolumab	II	Recurrent WHO grade 4 glioma	Recruiting	N/A	N/A	NCT04145115
	Ipilimumab and nivolumab *vs.* TMZ	II/III	Newly diagnosed MGMT unmethylated GBM	Active, not recruiting	N/A	N/A	NCT04396860
	Oncolytic HSV-1 G207	I	Pediatric progressive/recurrent HGG	For 12 patients, median OS 12.2 months	2021	^8^	NCT02457845
	G47Δ	I/II	Recurrent GBM	Median OS 7.3 months	2022	^12^	UMIN000015995
	DNX-2401 OV	I	Recurrent HGG	For 37 patients including 33 with GBM: median OS 13.0 months	2018	^14^	NCT00805376
	Intratumoral delivery of DNX-2401 OV followed by pembrolizumab	I/II	Recurrent GBM	For 49 patients including 48 with GBM: median OS 12.5 months, ORR 10.4%	2023	^15^	NCT02798406
	OH2 OV	I	Recurrent CNS malignant tumors	Recruiting	N/A	N/A	NCT05235074
	DCVax-L + TMZ (as SOC) *vs.* placebo (unmanipulated peripheral blood mononuclear cells) + TMZ (as SOC)	III	Newly diagnosed and recurrent GBM	For 232 patients with newly diagnosed GBM receiving DCVax-L: median OS 22.4 months (compared with 19.3 months); for 64 patients with recurrent GBM receiving DCVax-L: 42% relative risk reduction in the likelihood of death at any point	2023	^20^	NCT00045968
	Intramuscular CGA injection	I	Recurrent HGG	For 17 patients with WHO 4 glioma (including 15 GBM): median OS 9.5 months	2023	^23^	CTR20160113 NCT02728349
	Intramuscular CGA injection	II	Recurrent GBM	Recruiting	N/A	N/A	NCT03758014
Targeted therapy	Orally administered PAC-1 on days 1–21, with TMZ 150 mg/m^2^/5 days, per 28-day cycle	I	Recurrent HGG	Confirmed PR in 2 of 13 patients with GBM	2023	^25^	NCT03332355
	CDK4/6 inhibitor (auceliciclib) with TMZ	I	GBM	Recruiting	N/A	N/A	ACTRN12621000479808
	Regorafenib (160 mg once daily for the first 3 weeks of each 4-week cycle) or CCNU (110 mg/m^2^ every 6 weeks)	II	Recurrent GBM	For patients receiving regorafenib: median OS 7.4 months; for patients in the lomustine group: median OS 5.6 months	2019	^29^	NCT02926222
	Regorafenib	II/III	Newly diagnosed and recurrent GBM	Recruiting	N/A	N/A	NCT03970447
	Dabrafenib (150 mg orally twice daily) and oral trametinib (2 mg orally once daily)	II	Recurrent or progressive BRAFV600E mutant glioma	In the GBM cohort: ORR 32%; median OS 13.7 months	2022	^31^	NCT02034110
	Cohort 1: buparlisib before re-surgery; cohort 2: buparlisib only	II	PI3K pathway-activated GBM at first or second recurrence	Median follow-up 15.6 months in cohort 1 *vs.* 9.8 months in cohort 2	2019	^35^	NCT01339052
	Cohort 1: buparlisib (80/100 mg once daily) plus carboplatin (every 3 weeks); cohort 2: buparlisib (60 mg once daily) plus CCNU (every 6 weeks)	Ib/II	Recurrent GBM	Median PFS 1.4 months in cohort 1 *vs.* 1.3 months in cohort 2, indicating insufficient antitumor activity of buparlisib	2020	^36^	NCT01934361

CCNU, lomustine; GBM, glioblastoma; PFS, progression-free survival; OS, overall survival; ORR, overall response rate; HGG, high-grade gliomas; TMZ, temozolomide; OV, oncolytic viruses; DC, dendritic cell; SOC, standard of care; CGA, 5-caffeoylquinic acid; PR, partial response; PFS-6, PFS at 6 months.

## Discussion

Before the classification of gliomas based on molecular and genomic features, gliomas were classified into 4 grades based solely on morphological diagnoses. For instance, the diagnosis of GBM included both IDH mutation and wild type. However, diffuse gliomas are heterogeneous tumors, both histologically and molecularly. The grading has changed because of continued improvements in molecular profiling. The WHO 2021 classification restricts the term “glioblastoma” to IDH wild-type tumors, and the changes in the definition may potentially affect outcomes, thus obscuring the true effectiveness of treatments. Assessing and analyzing the heterogeneity of clinical trials is crucial to obtain reliable results.

Clinical glioma drug research faces hopes and challenges. Immunotherapy and targeted therapy offer hope to patients with glioma, but substantial barriers must still be overcome, including the challenges of BBB penetration and the need for personalized treatments based on complex molecular phenotypes. Well-designed preclinical studies and clinical trials with standardized processes and reliable data are essential for the successful development of new therapies that will ultimately benefit a broad range of patients.

Another important aspect of glioma drug research is exploring the potential of combined and systematic therapies to overcome drug resistance and maximize clinical benefits. With continuing advancements in molecular biology, genomics, and drug development technologies, progress is expected to be achieved for glioma treatment, thus offering patients improved survival through innovative systematic therapies.

Although the Stupp regimen has provided survival benefits for patients with HGG, particularly those with recurrent or deep-seated lesions that are difficult to operate on, disease management remains unclear and is influenced by multiple factors. Hence, NCCN guidelines encourage patients with HGG to participate in clinical trials if they are eligible. The increasing number of clinical trials is expected to lead to a wide range of therapeutic options for this lethal disease in the future.
